# Measurement and Isolation of Thermal Stress in Silicon-On-Glass MEMS Structures

**DOI:** 10.3390/s18082603

**Published:** 2018-08-08

**Authors:** Zhiyong Chen, Meifeng Guo, Rong Zhang, Bin Zhou, Qi Wei

**Affiliations:** Department of Precision Instruments, Tsinghua University, Beijing 100084, China; rongzh@tsinghua.edu.cn (R.Z.); zhoub@tsinghua.edu.cn (B.Z.); weiqi@tsinghua.edu.cn (Q.W.)

**Keywords:** microelectromechanical system (MEMS), silicon-on-glass, thermal stress, stress measurement, stress isolation

## Abstract

The mechanical stress in silicon-on-glass MEMS structures and a stress isolation scheme were studied by analysis and experimentation. Double-ended tuning forks (DETFs) were used to measure the stress based on the stress-frequency conversion effect. Considering the coefficients of thermal expansion (CTEs) of silicon and glass and the temperature coefficient of the Young’s modulus of silicon, the sensitivity of the natural frequency to temperature change was analyzed. A stress isolation mechanism composed of annular isolators and a rigid frame is proposed to prevent the structure inside the frame from being subjected to thermal stresses. DETFs without and with one- or two-stage isolation frames with the orientations <110> and <100> were designed, the stress and natural frequency variations with temperature were simulated and measured. The experimental results show that in the temperature range of −50 °C to 85 °C, the stress varied from −18 MPa to 10 MPa in the orientation <110> and −11 MPa to 5 MPa in the orientation <100>. For the 1-stage isolated DETF of <110> orientation, the measured stress variation was only 0.082 MPa. The thermal stress can be mostly rejected by a stress isolation structure, which is applicable in the design of stress-sensitive MEMS sensors and actuators.

## 1. Introduction

Mechanical stress is often an important factor that affects the performance of microelectromechanical system (MEMS) devices. Some types of sensors are based on stress transformation effects such as piezoresistive accelerometers [1,2], gyroscopes [3], pressure sensors [4,5,6], and piezoelectric sensors [7]. In these cases, the thermal stress in the structure leads directly to an output error. In other types of sensors and actuators, mechanical stresses can cause variations in the geometry of the element. For example, in a force-rebalance capacitive accelerometer, when the stress in asymmetric springs of the structure causes a displacement of the proof mass [8], the control circuit will generate an electrostatic voltage to correct the offset, resulting in a zero-bias deviation. In microelectromechanical angular rate gyroscopes, the stiffness of the suspension in certain directions can be altered when the position of the proof mass is shifted due to stress, so that the coupling signal and the zero-bias also change.

Generally, temperature is the main factor that causes the structural stress to change. Various materials such as silicon and glass have unequal coefficients of thermal expansion (CTEs), so that the internal stress of a structure formed by bonding different materials varies with temperature [9]. The CTE of the material may also vary with temperature; thus, the relationship between thermal stress and temperature is normally nonlinear. Therefore, the zero-bias deviation of a MEMS sensor is usually nonlinear with temperature.

There are several commonly used methods for measuring the stress in various MEMS structures. The first method is to measure the deformation caused by stress. The structures may buckle in or out of their plane, and the deformation can be estimated by directly observing the variation in the alignment point in the structure using an optical profiler [10], or by measuring the resistances between the deformed beam and the adjacent electrodes to determine which electrode it is in contact with [11]. The second method consists of testing the structure using Raman spectroscopy. During laser irradiation of the structure, the spectral frequency of the scattered light varies with stress. By comparing the spectroscopy with that of the unstressed structure, the internal stress at each point of the structure can be determined [12,13]. The third method utilizes piezoresistivity. To fabricate stress-sensitive patterns, piezoresistive material was doped into the surface of the structure. The stress in the structure causes the resistance to vary and the value of the stress can be determined by calculating the measured resistance of the patterns [14]. The fourth method uses the stress–frequency conversion. The natural frequency of a doubly clamped beam changes with the axial stress in the beam and can be measured using a scanning laser Doppler vibrometer system or frequency sweeping instruments, and then the stress can be determined [15,16].

In this article, the double-ended tuning fork (DETF) is adopted to detect the stress in a silicon-on-glass (SOG) MEMS structure. The relationships among the natural frequency of the DETF and the thermal expansion and elastic modulus thermal change of the materials are analyzed. The microstructures were fabricated, and by testing the natural frequencies of the DETF at different temperatures, the relationship between the stress in the structure and temperature was obtained.

The design of heat-insensitive structures or stress-isolating structures [17] are two direct ways to improve the performance of MEMS devices. To reduce stress in the MEMS structure and minimize its temperature change, this paper proposes a stress isolation structure that isolates the component core and anchors. To test the effect of stress isolation, the natural frequency of the DETF with the stress-isolating structure is measured.

## 2. Stress Testing Structure

### 2.1. Principle of Stress Test Structure

**Figure 1:**
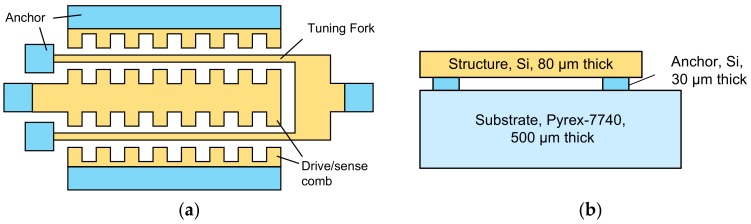
Schematic drawing of the stress test structure. (**a**) Top view; (**b**) the layer stack of the structure.

The stress test structure is designed based on the effect that the natural frequency of the DETF varies with the axial stress of the beam, and is realized by SOG microprocessing. The structural layer is phosphor-doped monocrystalline silicon with a concentration of 3 × 10^19^/cm^3^, the substrate is Pyrex 7740 glass, and anodic bonding combines the two. The schematic of the stress test structure is shown in Figure 1. The DETF is U-shaped, and the ends of the resonant beams are anchored to the glass substrate. The comb electrodes are arranged on both sides of the beam to drive it with electrostatic force and to sense vibration by detecting the change in capacitance between the electrode and the beam.

In the condition that the beam is not subjected to an axial force, the first-order natural frequency of the resonant beam is

(1) $$f_{0}=a^{2}/\left(2\pi l^{2}\right)\sqrt{EI/\left(\rho A\right)},$$

where *E* is the Young’s modulus, *ρ* is the density of the beam material, *l* is the length, *A* is the area of the section, *I* is the moment of inertia of the section of the beam, and *a* is a constant approximately equal to 4.73 [18,19,20].

As the temperature changes, the structural dimensions and the elastic moduli of silicon and glass vary, resulting in an axial stress of the silicon resonant beam, and therefore, its natural frequency. Subjected to an axial force *N*, the natural frequency of the resonant beam is

(2) $$f=f_{0}\sqrt{1+cNl^{2}/EI},$$

where *c* = 0.0245775 [19].

From (2), the axial stress *σ* is a function of the natural frequency:

(3) $$\sigma =\frac{Eb^{2}}{12cl^{2}}\left[{\left(f/f_{0}\right)}^{2}-1\right],$$

where *b* is the width of the beam.

Therefore, the structural stresses at various temperatures can be calculated from the results of the natural frequency measurements.

### 2.2. Natural Frequency Sensitivity to Temperature

When the temperature changes, the dimensions of the structure will change due to a thermal expansion effect. Since silicon and glass have unequal CTEs, the beam withstands axial force. In addition, Young’s modulus also changes with the temperature. Refer to (1) and (2), the axial force and the dimensions and the Young’s modulus of the DETF, they are all factors that affect the natural frequency. In order to determine which factor is the most significant, it is necessary to analyze the sensitivity of the natural frequency to the temperature.

#### 2.2.1. Stress Analysis

Due to the complexity of the thermal deformation of the whole structure, it is almost impossible to obtain an accurate stress distribution by an analytical method. Based on the assumption that the stresses in the glass substrate and the silicon beam are both uniformly distributed, we made an approximate analysis of the structural stress.

Suppose that the structure has no internal stress at temperature *T*_0_; then, the temperature variation is defined as Δ*T* = *T* − *T*_0_, where *T* is the temperature. At a temperature close to *T*_0_, assume that the CTEs of silicon and glass are both constant. As the force and the reaction force between the silicon beam and glass substrate are equal,

(4) $$E_{Si}A_{Si}\frac{l_{bond}-l_{b0}\left(1+\alpha_{Si}\Delta T\right)}{l_{b0}\left(1+\alpha_{Si}\Delta T\right)}=-E_{G}A_{G}\frac{l_{bond}-l_{b0}\left(1+\alpha_{G}\Delta T\right)}{l_{b0}\left(1+\alpha_{G}\Delta T\right)},$$

where *l_bond_* is the distance between the anchors, *l_b_*_0_ is the *l_bond_* at *T*_0_, *A_Si_* and *A_G_* are the section areas of the beam and substrate, respectively, *E_Si_* and *E_G_* are the Young’s moduli of silicon and glass, respectively, and *α_Si_* and *α_G_* are the CTEs of silicon and glass, respectively.

Then,

(5) $$l_{bond}=\frac{\left(E_{G}A_{G}+E_{Si}A_{Si}\right)l_{b0}\left(1+\alpha_{G}\Delta T\right)\left(1+\alpha_{Si}\Delta T\right)}{E_{Si}A_{Si}\left(1+\alpha_{G}\Delta T\right)+E_{G}A_{G}\left(1+\alpha_{Si}\Delta T\right)}.$$

*E_Si_* and *E_G_* are of the same order of magnitude (130 GPa and 169 GPa in the <100> and <110> orientations, respectively, for silicon, and 63 GPa for Pyrex 7740), whereas the transverse substrate surface is much larger than that of the silicon beam; therefore,

(6) $$l_{bond}\approx l_{b0}\left(1+\alpha_{G}\Delta T\right).$$

The distance between the anchor points is mainly affected by the CTE of the glass. Hence, the axial force in the beam is

(7) $$N=E_{Si}A_{Si}\left[\frac{l_{b0}\left(1+\alpha_{G}\Delta T\right)}{l_{b0}\left(1+\alpha_{Si}\Delta T\right)}-1\right]\approx E_{Si}A_{Si}\left(\alpha_{G}-\alpha_{Si}\right)\Delta T.$$

Over a wide temperature range, the CTEs of silicon and Pyrex 7740 vary with temperature, and

(8) $$N=E_{Si}A_{Si}\int_{T0}^{T}\left(\alpha_{G}-\alpha_{Si}\right)dT.$$

The difference between the CTEs of glass and silicon is

(9) $$\alpha_{D}=\alpha_{G}-\alpha_{Si}.$$

After the simplification of *E_Si_* and *A_Si_* to *E* and *A*, respectively, the axial force is

(10) $$N=EA\int_{T0}^{T}\alpha_{D}dT,$$

the axial stress is

(11) $$\sigma =E\int_{T0}^{T}\alpha_{D}dT.$$

#### 2.2.2. Natural Frequency Variation with Temperature

The CTE of silicon at 20 °C is approximately 2.6 × 10^−6^/°C, and the CTE of Pyrex 7740 is 3.25 × 10^−6^/°C in the temperature range of 0 to 300 °C [21,22,23].

**Figure 2:**
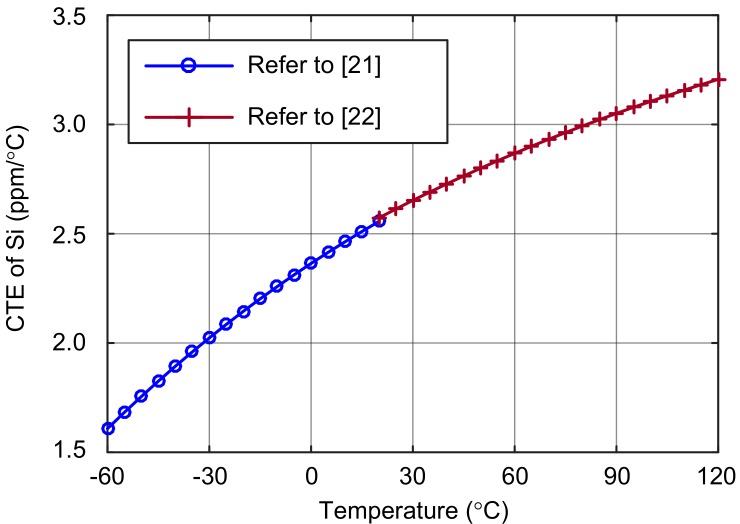
The coefficient of thermal expansion of silicon.

Authors of two previous papers [21,22] measured the CTE of silicon at −266–20 °C and 20–1000 °C, respectively. By combining their measurement results, the CTE of silicon in the temperature range of −60–125 °C is shown in Figure 2.

The authors of two previous papers [24,25] provided the Young’s moduli of silicon in various crystal orientations. The Young’s moduli have different temperature coefficients depending on the orientation; a past study [26] reported the temperature coefficients for the Young’s modulus of P-type silicon: *Tc*_44_ = −60.14 ppm/°C, *Tc*_12_ = −91.59 ppm/°C, and *Tc*_11_ = −73.25 ppm/°C. In this article, we use the symbol “*β*” instead of “*Tc*”.

From (1) to (3) and (11), the natural frequency of the tuning fork is

(12) $$f=\frac{a^{2}}{2\pi l^{2}}\sqrt{\frac{EI}{\rho A}}\sqrt{1+12c\frac{l^{2}}{b^{2}}\int_{T0}^{T}\alpha_{D}dT}=R_{1}R_{2}R_{3},$$

where

(13) $$R_{1}=a^{2}/\left[2\pi l_{0}^{2}{\left(1+\int_{T_{0}}^{T}\alpha_{Si}dT\right)}^{2}\right],$$

(14) $$R_{2}=\sqrt{\left[E_{0}\left(1+\int_{T_{0}}^{T}\beta dT\right)h_{0}b_{0}^{3}l_{0}{\left(1+\int_{T_{0}}^{T}\alpha_{Si}dT\right)}^{5}\right]/\left(12m\right)},$$

(15) $$R_{3}=\sqrt{1+12c{\left(l_{0}/b_{0}\right)}^{2}\int_{T0}^{T}\alpha_{D}dT},$$

*b*_0_, *h*_0_, *l*_0_, and *E*_0_ are the width, thickness, length of the beam, and Young’s modulus along the length of the beam at *T*_0_, respectively, and *m* is the mass of a single arm of the tuning fork.

The natural frequency sensitivity to temperature:

(16) $$\frac{df}{dT}=\frac{\partial f}{\partial R_{1}}\cdot \frac{dR_{1}}{dT}+\frac{\partial f}{\partial R_{2}}\cdot \frac{dR_{2}}{dT}+\frac{\partial f}{\partial R_{3}}\cdot \frac{dR_{3}}{dT}.$$

As *α_Si_* and *β* are on the order of 10^−6^/°C and 10^−4^/°C,

(17) $$\begin{array}{cc} \frac{df}{dT} & \approx \left(-R_{2}R_{3}\frac{a^{2}}{\pi l_{0}^{2}}+R_{1}R_{3}\frac{5E_{0}I_{0}l_{0}}{2mR_{2}}\right)\alpha_{Si}+R_{1}R_{3}\frac{E_{0}I_{0}l_{0}}{2mR_{2}}\beta +\frac{R_{1}R_{2}}{R_{3}}\cdot \frac{6cl_{0}^{2}}{b_{0}^{2}}\alpha_{D}\\ & \approx R_{1}R_{3}\frac{E_{0}I_{0}l_{0}}{2mR_{2}}\left(\alpha_{Si}+\beta \right)+\frac{R_{1}R_{2}}{R_{3}}\cdot \frac{6cl_{0}^{2}}{b_{0}^{2}}\alpha_{D} \end{array}$$

(18) $$R_{1}\approx a^{2}/\left(2\pi l_{0}^{2}\right),$$

(19) $$R_{2}\approx \sqrt{E_{0}h_{0}b_{0}^{3}l_{0}/\left(12m\right)}=\sqrt{E_{0}b_{0}^{2}/\left(12\rho_{0}\right)},$$

where *ρ*_0_ is the density of silicon at *T*_0_.

For the proposed structure, *l*_0_, *b*_0_ and *h*_0_ are 1200 μm, 7 μm and 80 μm respectively. The value of *R*_3_ is 0.55 at −60 °C and 1.3 at 125 °C. At a temperature of approximately *T*_0_, *R*_3_ ≈ 1, so

(20) $$\frac{df}{dT}\approx \frac{a^{2}}{2\pi }\sqrt{\frac{3E_{0}}{\rho }}\left[\frac{b_{0}}{12l_{0}^{2}}\left(\alpha_{Si}+\beta \right)+\frac{c}{b_{0}}\cdot \alpha_{D}\right].$$

For the DETF with <110> orientation in this design,

(21) $$\frac{df}{dT}\approx 2.1278\times 10^{4}\left(\alpha_{Si}+\beta \right)+1.8442\times 10^{8}\alpha_{D}.$$

Since *α_Si_* and *α_D_* are on the order of 10^−6^/°C and since *β* is close to 10^−4^/°C, the main factor of the natural frequency variation is the CTE mismatch between silicon and glass. According to (21), the sensitivity of frequency to temperature caused by CTE mismatch, Silicon’s Young’s modulus and CTE of silicon is 120, 1.5, and 0.052 Hz/°C, respectively at *T*_0_. The primary factor causing the frequency change with temperature is the mismatch of CTE, the secondary factor is the change of Young’s modulus, and finally the change of the beam’s geometry size.

### 2.3. Stress and Natural Frequency Simulations

We constructed the finite element analysis model of the stress test structure in ANSYS. The stress and natural frequency variations with temperature are simulated for the beams with <100> and <110> orientations, respectively. In the simulation, the temperature range is set to −55–90 °C. The thermal expansions of silicon and glass are considered, but the variation in the elastic moduli with temperature was not taken into account. The structure is assumed to have no internal stress at 20 °C. First, thermal simulation is carried out to obtain the stress distribution in the structure, and then the stress distribution is transferred to the modal simulation phase. By the modal simulating with prestress effect, the vibration modes and corresponding natural frequencies of DETF at different temperatures are obtained.

For the resonance beam of orientation <110>, the simulated axial stress distribution is uniform over most parts of the beam, except for the regions adjacent to the two ends. The axial stress at the geometric center of the beam was taken as the representative of the internal stress.

**Figure 3:**
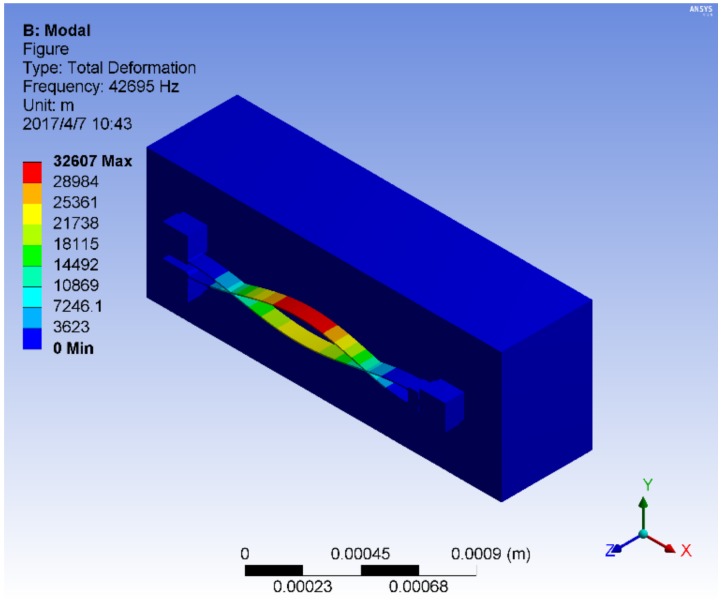
The first differential vibration mode of the double-ended tuning fork (DETF).

Figure 3 shows the simulated first differential vibration mode. For DETFs with crystal orientations <110> and <100>, the corresponding natural frequencies at 20 °C are 42,713 Hz and 35,447 Hz, respectively.

## 3. Stress Isolation

In the SOG structure, the glass layer is much thicker than the silicon layer and has greater rigidity. In the event of a change in temperature, the silicon layer is forced to elongate or compress with the glass. By designing a deformable structure to isolate the thermal expansion of the two layers, it is possible to reduce the stress in the silicon structure.

### 3.1. Stress Isolator

**Figure 4:**
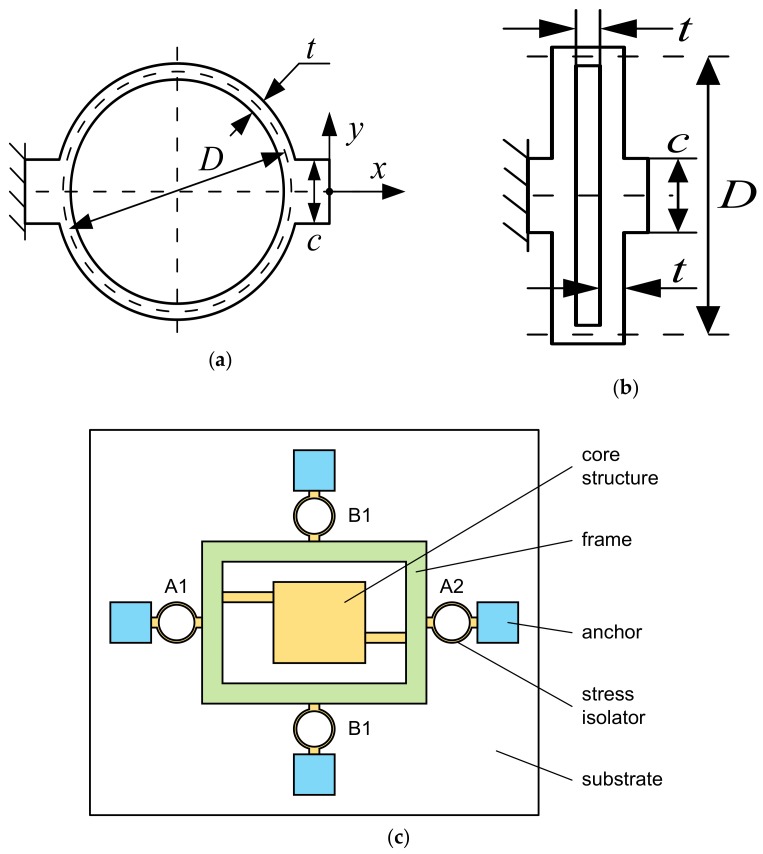
Two types of thermal stress isolator. (**a**) Annular isolator; (**b**) rectangular isolator; and (**c**) the usage of the isolators.

We designed two types of thermal stress isolators, which are annular and rectangular, respectively, as shown in Figure 4a,b. They can be used to isolate the thermal deformation mismatch of the silicon and glass in the *x* direction. Figure 4c illustrates the use of stress isolators in a MEMS structure. The core of the sensor or actuator is suspended in a rigid frame, and the frame is connected to the anchors by stress isolators.

Due to the flexibility of the isolators and rigidity of the frame in the *x* direction, the deformation of the entire structure at different temperatures occurs mainly on the isolators. Since the material of both the frame and core is silicon, ideally, there is almost no internal stress at any temperature. In reality, the extent of the stress in the core structure, or the effect of stress isolation, depends on the layout and parameters of the isolators and the frame.

During the design process of the structure, the stiffness of the isolator, which depends on its geometric dimensions, must be determined. However, there is no theoretical solution for the stiffness of an anisotropic structure, and finite element analysis is required. Referring to Figure 4a,b, in the stiffness simulation, the left end of the isolator is fixed, and a uniformly distributed force is applied to the right end in the *x* or *y* direction. The displacements of the centroid of the right end face in the *x* and *y* directions were taken as the deflections of the isolator. By dividing the applied force by the deflections, we obtained the stiffness of the annular and rectangular isolators in the *x* and *y* directions. Table 1 shows the dimensions of the isolators and the corresponding stiffness.

From the data in Table 1, it can be seen that for the same *D* and *t* values, the stiffness in the *x* direction (or primary stiffness, *K_x_*) of the annular isolator is lower than but close to that of the rectangular isolator. The stiffness in the *y* direction (or secondary stiffness, *K_y_*) of the annular isolator is less than its primary stiffness by an order of magnitude, whereas the secondary stiffness of the rectangular isolator is in the same order of magnitude as its primary stiffness.

A rectangular isolator has an advantage over an annular isolator in that it occupies a smaller area to achieve a certain stiffness. However, the use of annular isolators can simplify the design of the structural parameters. For example, in the case of Figure 4c, stress isolation is required in both the *x* and *y* directions, so several annular isolators are arranged around the frame. Isolators A1 and A2 are used to isolate stress in the *x* direction, and B1 and B2 are to isolate stress in the *y* direction. Since the secondary stiffness of an annular isolator is much lower than the primary stiffness, the *x* direction stiffness of the suspension is slightly affected by B1 and B2 and mainly determined by isolators A1 and A2. In the *y* direction, the opposite is true. The influence of the secondary stiffness can be neglected so it is easier to determine the isolator parameters.

### 3.2. DETF with Isolation Structure

**Figure 5:**
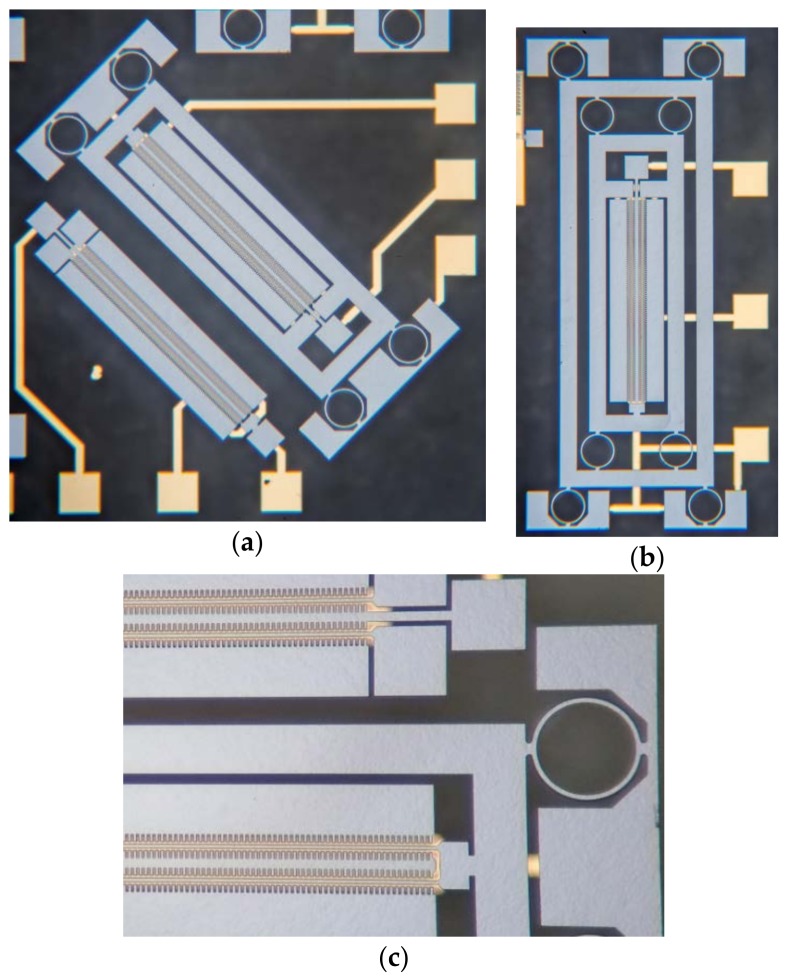
The DETFs and stress isolation structures. (**a**) Stress test structures of orientation <100> with and without stress isolation; (**b**) stress test structure of orientation <110> with 2-stage stress isolation; and (**c**) the details of the DETF structure of orientation <110>.

To verify the effect of stress isolation, five structures were designed: DETFs with crystal orientations <100> and <110> without stress isolation, DETFs with orientations <100> and <110> suspended in a 1-stage stress-isolated frame, a DETF of orientation <110> suspended in a 2-stage stress-isolated frame. All DETFs are identical. The photos of the structures are shown in Figure 5.

The frame width is 100 μm, ensuring the rigidity of the rectangular frame. The isolators are arranged along the axis of the frame beams because of the high stiffness during the stretching of the beams. Such an arrangement minimizes the deformation of the frame caused by thermal stress; therefore, the residual stress in the resonant beam is minimized.

## 4. Experiment

### 4.1. Test System

**Figure 6:**
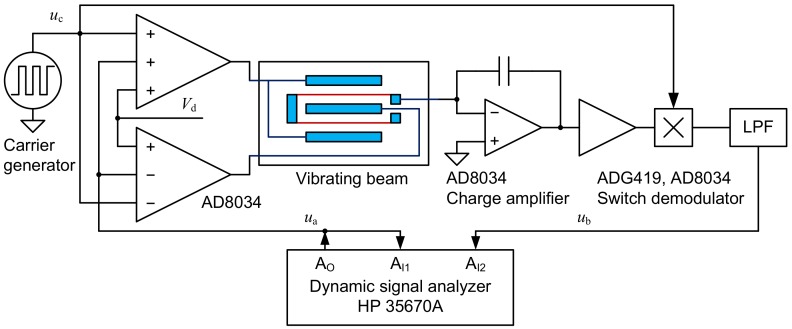
Test circuit and apparatuses.

To test the proposed structures, a drive and detection circuit is designed. The experimental system is composed of the circuit and a dynamic signal analyzer, as illustrated in the diagram in Figure 6. A pair of differential voltages is applied to the drive/sense combs to generate an electrostatic force. The vibration causes the capacitance between the combs and beam to vary, and the capacitance is converted into an amplitude-modulated square wave by a charge amplifier. The signal is amplified and then demodulated by the carrier. A low-pass circuit filters the demodulated signal and outputs the beam vibration signal.

A Hewlett-Packard HP35670A dynamic signal analyzer was used to generate the drive signal, and both the drive and the vibration signals are fed to the instrument. The frequency response characteristic of the beam was tested via frequency sweep experiments.

### 4.2. Test Method

The test samples are packaged in leadless ceramic chip carriers that are soldered to a FR4-based PCB, and the PCB is mounted in a temperature chamber. The experiments are carried out from −50–85 °C. The temperature was adjusted to change in steps: 25, 40, 55, 70, 85, 70, 55, 40, 25, 5, −15, −35, −50, −35, −15, 5, and 25. After the indicated temperature was stabilized at each set point for 45 min, we began the frequency sweeping experiments.

The frequency at which the amplitude–frequency curve reaches its peak is considered the resonant frequency *f_r_*. The quality factor of the vibrating beam can be estimated by dividing the peak amplitude *A_r_* by the low-frequency amplitude *A*_0_:

(22) $$Q\approx A_{r}/A_{0}.$$

The damping ratio is

(23) ζ=1/(2Q).

The natural frequency can be calculated by

(24) $$f_{0}=f_{r}/\sqrt{1-2\zeta^{2}}.$$

In the experiments, *A*_0_ was taken from the amplitude at 3 kHz.

### 4.3. Stress of the SOG Structure

**Figure 7:**
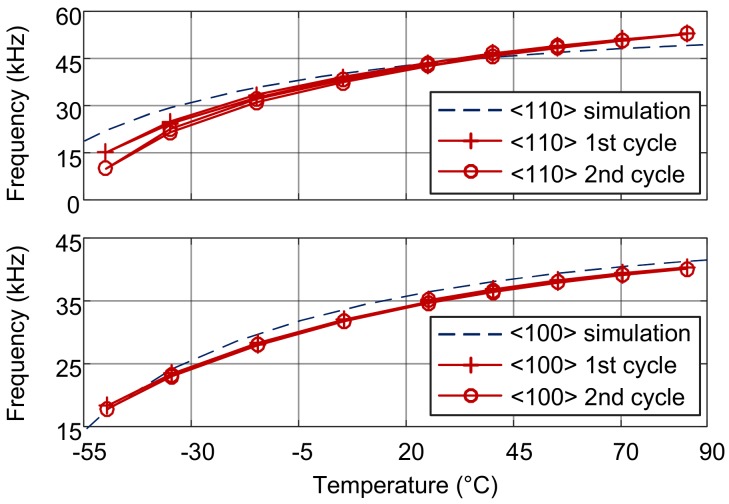
Natural frequency of the DETFs without stress isolation.

**Figure 8:**
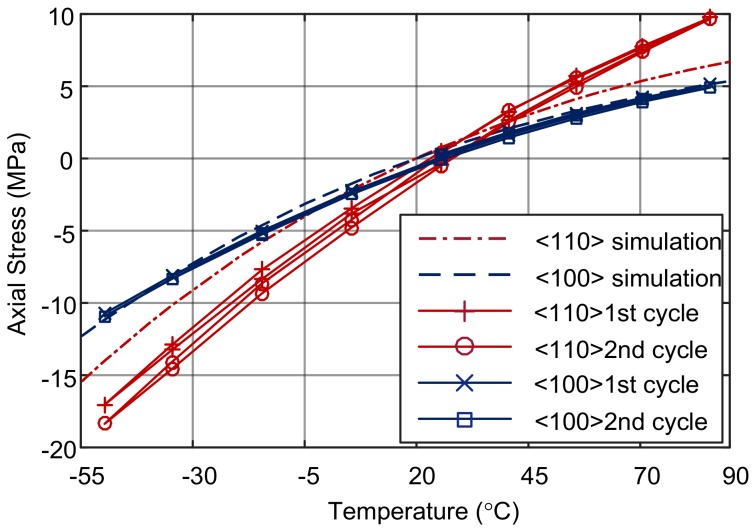
Axial stress of the DETFs without stress isolation.

The simulated and measured natural frequency-temperature relationships of the DETFs without isolation are illustrated in Figure 7. Depending on the manufacturing process of the microstructure, it is assumed that the stress in the DETFs is near zero at 20 °C. Based on the measured frequency data, the actual stress is estimated according to the frequency-stress relationship (3). Figure 8 illustrates the measured and simulated stress-temperature relationships of the DETFs fabricated with the SOG technology.

The results of the simulations and experiment show that the axial stress in the resonant beam has a nonlinear relationship with temperature. The axial stress is tensile at a temperature above *T*_0_. The stress and natural frequencies increase with increasing temperature, but the rate of increase slows down. At a temperature below *T*_0_, the axial stress is compressive. As the temperature decreases, the stress and natural frequency decreases more rapidly. This is because the difference between the CTEs of glass and silicon (*α_D_*) in this temperature range increases as the temperature decreases.

According to the simulation, the stresses in the surface orientations <100> and <110> are 3.93 and 4.93 MPa, respectively, at 85 °C, and −10.04 and −12.59 MPa, respectively, at −50 °C. In the temperature range of 135 °C the stress vary by 14.0 MPa and 17.5 MPa in <100> and <110> orientations, respectively.

However, the experiments show that the stress in orientation <100> varied from about −11 to 5 MPa, and the stress in orientation <110> varied from about −18 to 10 MPa from −50 to +85 °C. The measured stress variations are about 15% greater in the orientation <100> and 50% greater in the orientation <110>, respectively, than those of the simulation results. The gap between them can probably attributed to the boundary conditions of the simulation. In fact, the entire bottom of the glass substrate is glued to a ceramic carrier, but in the simulation, only a very small part of the bottom of the substrate is fixed, making it possible to relax the substrate and the DETFs. Also, the ceramic chip carrier was not modeled and therefore the influence of its thermal expansion was not reflected in the simulation. The stress difference between the experiment and the simulation shows the stress effect of the package.

### 4.4. Effect of Stress Isolation

**Figure 9:**
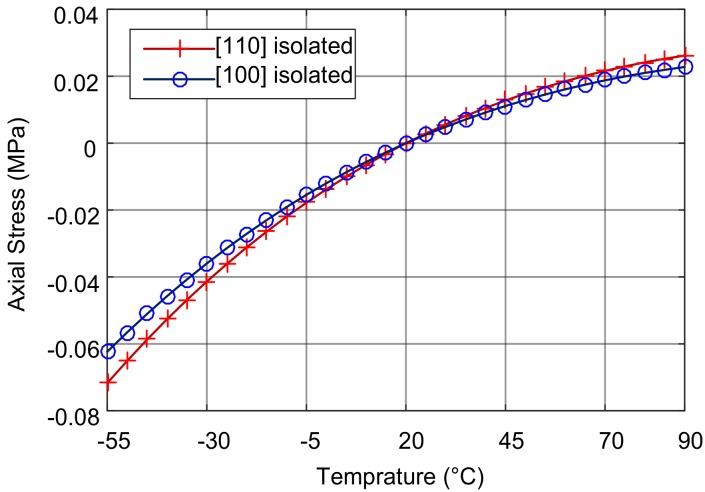
Axial stress of 1-stage isolated DETF (Simulation).

The simulation data of stress variation with temperature for DETF with a 1-stage stress isolation structure is shown in Figure 9. The axial stress in the 1-stage-isolated DETF with the orientations <110> and <100> vary only about 0.1 MPa and 0.085 MPa, respectively, in the temperature range of −50 to +85 °C. Comparing to the stress simulation result of the DETFs without stress isolation (17.5 MPa and 14.0 MPa with the orientations <110> and <100>, respectively), 99.4% of the thermal stress can be suppressed using the 1-stage isolation structure.

**Figure 10:**
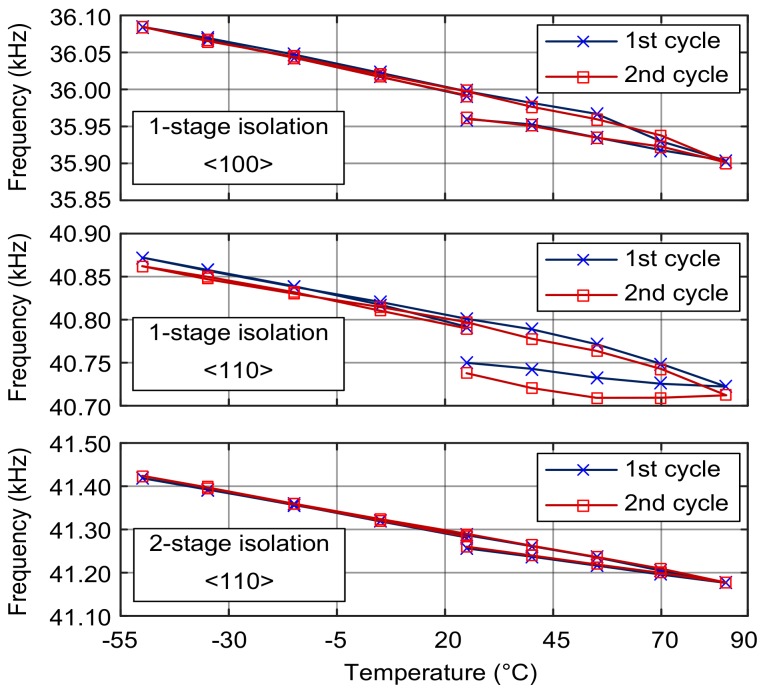
Natural frequency of the stress-isolated DETFs.

The experimental data on the frequencies of the DETFs with the stress isolation structure as a function of temperature are shown in Figure 10. They only change 0.2–0.3 kHz in the temperature range of −50–85 °C, which is far below the measured frequency variation of 22–40 kHz of the DETFs without the stress isolation structure (Figure 7).

Hysteresis in the frequency variation with temperature can be seen in Figure 10, which means there are hysteresis in the stress of the tuning fork. We suspect that the stress hysteresis may be due to the temperature characteristics of the glue that bonds the DETF chips to the ceramic carrier or the temperature characteristics of the PCB.

The natural frequency of the DETF without stress isolation increases with temperature, whereas that of the 1-stage isolated DETF decreases. This result shows that the 1-stage stress isolation structure attenuates most of the stress caused by the mismatch of the CTEs. The negative slope between frequency and temperature is mainly due to the negative temperature coefficient of the modulus of elasticity of silicon. The effect of stress isolation is significant.

In Figure 10, the 2-stage isolated DETF has a stronger tendency than the 1-stage isolated DETF, indicating that there was still a residual stress in the 1-stage isolated vibrating beam. Additionally, the frequency repeatability of the 2-stage isolated DETF is better than the 1-stage isolated structure. We considered that the difference between the frequency relative variations of the 1-stage and 2-stage isolated structures represents the residual stress in the 1-stage isolated beam.

On the basis of the data presented in Figure 10, the relative frequency variations of the 1-stage and 2-stage isolated DETFs with orientation <110> are −0.367% and −0.593%, respectively, from −50 °C to 85 °C. These data imply that the frequency variation due to the stress in the 1-stage isolated DETF is 0.226%. According to (3), the stress variation of the 1-stage isolated DETF from −50 °C to 85 °C is 0.082 MPa. Based on the stress variation of 28 MPa in the DETF without the stress isolation structure of <110> orientation, we can claim that a single-stage stress isolation structure removes the thermal stress by 99.7%.

Therefore, the axial force is negligible in the 2-stage isolated DETF, and almost all the natural frequency changes are caused by the Young’s modulus variation in the silicon structure.

## 5. Conclusions

For the MEMS element fabricated using a SOG process, the mechanical stress in the structure results from the CTE mismatch between the silicon layer and the glass substrate. The natural frequency variation of a DETF is determined by the axial stress caused by CTE mismatch, the temperature coefficient of the Young’s modulus of the DETF material and the CTE caused dimension variation. The CTE mismatch dominates the frequency variation, the effects of the changes in the Young’s modulus, and the dimensions of silicon are 2 and 3–4 orders of magnitude less than the effect of the CTE mismatch, respectively. The analyses and simulations show nonlinear temperature-stress relationships. According to the experimental results, from −50 °C to 85 °C, the thermal stress in the silicon structure varies approximately by 28 MPa with orientation <110> and by 16 MPa with orientation <100>. By suspending the central structure of an SOG element in the proposed stress isolation structure composed of annular isolators and a rigid frame, the thermal stress can be mostly rejected. For the stress isolation structure with the dimensions given in this work, a single-stage isolation scheme can suppress the thermal stress by 99.7%. The stress isolation scheme is applicable in the design of stress-sensitive MEMS sensors and actuators.

## Figures and Tables

**Table 1:** Simulation values of the isolator stiffness.

Isolator Type	*D* (μm)	*t* (μm)	*K_x_* (N/m)	*K_y_* (N/m)
Annular	100	10	7.324 × 10^4^	6.547 × 10^3^
Annular	200	10	8.435 × 10^3^	9.031 × 10^2^
Annular	200	20	6.018 × 10^4^	4.137 × 10^3^
Rectangular	100	10	1.092 × 10^5^	8.131 × 10^4^
Rectangular	200	10	1.489 × 10^4^	4.454 × 10^4^
Rectangular	200	20	8.764 × 10^4^	5.543 × 10^4^

## Supplementary Materials

The following are available online at http://www.mdpi.com/1424-8220/18/8/2603/s1, Figure 1. Simulated frequency response of the DETF with orientation <110>; Table 1. Experimental natural frequencies of the stress test DETFs.
