# Supplementary material for: Measurement and Isolation of Thermal Stress in Silicon-On-Glass MEMS Structures
Source: Sensors (Basel). 2018 Aug 8;18(8):2603. doi: 10.3390/s18082603 (PMC6111565; doi:10.3390/s18082603)
Supplement: Supplementary file 1 [file sensors-18-02603-s001.pdf]

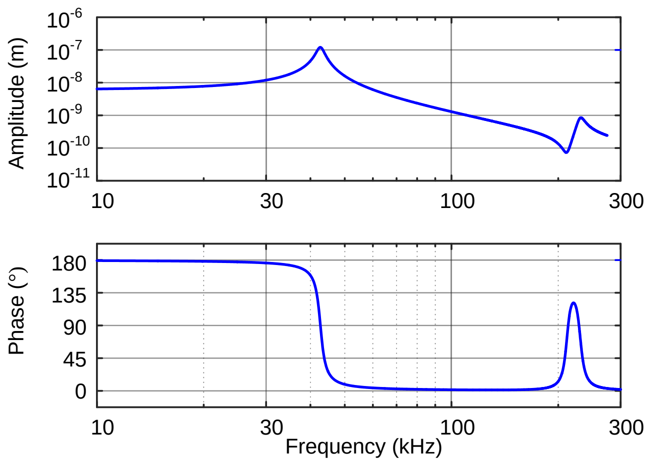

Figure 1. Simulated frequency response of the DETF

Table 1. Experimental natural frequencies of the stress test DETFs

Temperature (°C)	Natural frequency (kHz)				
	<100> without isolation	<100> 1-stage isolated	<110> without isolation	<110> 1-stage isolated	<110> 2-stage isolated
25	34.856	35.961	43.493	40.742	41.260
40	36.494	35.951	46.587	40.737	41.240
55	37.890	35.935	48.854	40.728	41.219
70	39.015	35.923	50.915	40.716	41.199
85	39.867	35.901	52.730	40.713	41.177
70	38.867	35.938	50.625	40.750	41.209
55	37.646	35.960	48.258	40.759	41.236
40	36.194	35.977	45.740	40.776	41.262
25	34.463	35.997	42.498	40.793	41.290
5	31.558	36.020	37.353	40.812	41.324
-15	27.883	36.044	30.941	40.831	41.360
-35	22.943	36.065	21.389	40.850	41.398
-50	17.776	36.085	9.710	40.862	41.424
-35	22.903	36.066	22.447	40.849	41.396
-15	27.774	36.043	31.999	40.831	41.358
5	31.544	36.017	38.036	40.809	41.321
25	34.492	35.992	43.283	40.790	41.283
